# MTHFD2 Blockade Enhances the Efficacy of β-Lapachone Chemotherapy With Ionizing Radiation in Head and Neck Squamous Cell Cancer

**DOI:** 10.3389/fonc.2020.536377

**Published:** 2020-11-11

**Authors:** Kirtikar Shukla, Naveen Singh, Joshua E. Lewis, Allen W. Tsang, David A. Boothman, Melissa L. Kemp, Cristina M. Furdui

**Affiliations:** ^1^Department of Internal Medicine, Section on Molecular Medicine, Wake Forest School of Medicine, Winston-Salem, NC, United States; ^2^Department of Biochemistry and Molecular Biology, Simon Cancer Center, Indiana University School of Medicine, Indianapolis, IN, United States; ^3^The Parker H. Petit Institute of Bioengineering and Bioscience, Georgia Institute of Technology, Atlanta, GA, United States; ^4^The Wallace H. Coulter Department of Biomedical Engineering, Georgia Institute of Technology and Emory School of Medicine, Atlanta, GA, United States

**Keywords:** head and neck cancer, radiation resistance, NQO1, β-lapachone, MTHFD2

## Abstract

Head and Neck Squamous Cell Cancer (HNSCC) presents with multiple treatment challenges limiting overall survival rates and affecting patients' quality of life. Amongst these, resistance to radiation therapy constitutes a major clinical problem in HNSCC patients compounded by origin, location, and tumor grade that limit tumor control. While cisplatin is considered the standard radiosensitizing agent for definitive or adjuvant radiotherapy, in recurrent tumors or for palliative care other chemotherapeutics such as the antifolates methotrexate or pemetrexed are also being utilized as radiosensitizers. These drugs inhibit the enzyme dihydrofolate reductase, which is essential for DNA synthesis and connects the 1-C/folate metabolism to NAD(P)H and NAD(P)^+^ balance in cells. In previous studies, we identified MTHFD2, a mitochondrial enzyme involved in folate metabolism, as a key contributor to NAD(P)H levels in the radiation-resistant cells and HNSCC tumors. In the study presented here, we investigated the role of MTHFD2 in the response to radiation alone and in combination with β-lapachone, a NQO1 bioactivatable drug, which generates reactive oxygen species concomitant with NAD(P)H oxidation to NAD(P)^+^. These studies are performed in a matched HNSCC cell model of response to radiation: the radiation resistant rSCC-61 and radiation sensitive SCC-61 cells reported earlier by our group. Radiation resistant rSCC-61 cells had increased sensitivity to β-lapachone compared to SCC-61 and knockdown of MTHFD2 in rSCC-61 cells further potentiated the cytotoxicity of β-lapachone with radiation in a dose and time-dependent manner. rSCC-61 MTHFD2 knockdown cells irradiated and treated with β-lapachone showed increased PARP1 activation, inhibition of mitochondrial respiration, decreased respiration-linked ATP production, and increased mitochondrial superoxide and protein oxidation as compared to control rSCC-61 scrambled shRNA. Thus, these studies point to MTHFD2 as a potential target for development of radiosensitizing chemotherapeutics and potentiator of β-lapachone cytotoxicity.

## Introduction

Head and neck squamous cell cancer (HNSCC) is an aggressive disease with a high rate of mortality and morbidity in the United States. A recent statistical report from the National Cancer Institute estimated that more than 53,000 people will have oropharyngeal cancer in the United States in 2020 accounting for ~4–5% of overall cancer types (1). Radiation therapy is a key component of standard of care treatment for HNSCC, but radioresistance and side effects of treatment limit patients' overall survival and long-term quality of life (2). There is a need for new molecular approaches to treat radiation resistant HNSCC tumors using combination therapies, and indeed, current efforts to enhance the efficacy of radiation therapy in HNSCC include combination treatment with targeted therapies (e.g., against Epidermal Growth Factor Receptor, EGFR), and immunomodulators, which are increasingly investigated in clinical studies (3).

Over the last decades, work pioneered by Boothman's group has established β-lapachone (β-lap), a NAD(P)H:quinone oxidoreductase 1 (NQO1) bioactivatable substrate, as a promising cancer therapeutic and radiation sensitizer targeting a broad spectrum of cancers including HNSCC (4–9). NQO1 metabolizes β-lap into an unstable hydroquinone, which is then oxidized back to the quinone state through a semiquinone intermediate releasing superoxide ${\text{O}}_{2}^{-}$. This futile redox cycle depletes the cells of NAD(P)H and generates toxic amounts of reactive oxygen species (ROS) leading to DNA damage and PARP1 hyperactivation (5, 6, 10, 11). PARP1 activity further consumes the NAD^+^ produced in the NQO1/β-lap cycle instigating a specific mechanism of μ-Calpain-mediated cell death called Keresis (12). The cumulative body of evidence supports the tumor-selective efficacy of β-lap derivatives and a version of this, ARQ761, has already been investigated in phase I/II clinical trials for patients with metastatic solid tumors (13). However, not all patients responded to treatment and while there was an association of tumor response with NQO1 expression, clearly other factors limited the efficacy of β-lap/ARQ761 treatment in these studies. Logically, based on the known mechanism of action, the cytotoxicity of β-lap is expected to be driven by the expression and activity of NQO1, the expression and activity of ROS-metabolizing enzymes (e.g., catalase, SOD1, etc.) (11, 14), and the availability of NAD(P)H, which is needed to support both the NQO1-catalyzed generation of ROS and the activity of key ROS-suppressing redox regulatory enzymes [e.g., thioredoxin reductase (14)]. In this context, the localization of NAD(P)H may also become important considering the predominant cytoplasmic localization of NQO1. The expression of NQO1 is regulated in cells by KEAP1/Nrf2 pathways, and a series of studies have established prolonged induction of NQO1 expression by ionizing radiation in lung cancer cells and in FSall mice tumors (15, 16). These findings raised the possibility of improved chemotherapeutic and radiation sensitizing activity of β-lap, which have now been confirmed in numerous cancers, including HNSCC (9).

Recognizing the importance of NAD(P)H availability to sustain the NQO1-dependent activity of β-lap, we have started to investigate the metabolic contribution to intracellular NAD(P)H first by computational flux balance analysis taking advantage of HNSCC data available in online repositories (e.g., TCGA, Human Protein Atlas), and multi-omics data collected for a matched model of response to radiation (radiation sensitive SCC-61 and radiation resistant rSCC-61) developed by our group (17–21).

In the study presented here, we evaluated mitochondrial methylenetetrahydrofolate dehydrogenase 2 (MTHFD2), one of the metabolic enzymes identified by the computational studies as a major producer of NAD(P)H in radiation resistant HNSCC cells and patient tumors. We report a series of *in vitro* and *in vivo* studies assessing the role of MTHFD2 in enhancing the efficacy of response to ionizing radiation and β-lap using the radiation sensitive SCC-61 and radiation resistant rSCC-61 matched cell system highlighted above.

## Materials and Methods

### Materials

The following materials were utilized for the studies included here: Dulbecco's Modified Eagle Medium/Nutrient Mixture F-12 (DMEM/F12), penicillin/streptomycin, fetal bovine serum (FBS) (Gibco, Thermo Fisher Scientific, USA); β-lap (Xoder Technologies, USA); Lipofectamine 2000 and oligomycin (Thermo Fisher Scientific, USA); carbonyl cyanide 4-(trifluoromethoxy) phenylhydrazone (FCCP) (Cayman Chemicals, USA); Antimycin A (Abcam, USA); Rotenone (Millipore-Sigma, USA); MitoSOX (Invitrogen, Thermo Fisher Scientific, USA); antibodies against NQO1, MTHFD2, catalase, PARP1, p-γH_2_AX(S139), β-actin, and GAPDH (Cell Signaling Technology, USA); shRNA (MTHFD2 and scrambled control), PAR and α-tubulin antibodies (Santa Cruz Biotechnology, USA); Bicinchoninic acid (BCA) assay, CyQuant kit, and SuperSignal chemiluminescent HRP substrate (Thermo Fisher Scientific, USA). Matrigel Growth Factor Reduced (GFR) Basement Membrane Matrix, LDEV-free was obtained from Corning Inc., USA (LDEV-free: free of viruses, including lactose dehydrogenase elevating virus or LDEV). Modified RIPA buffer for cell lysis was prepared in the laboratory and contained: 50 mM Tris-HCl, pH 7.4; 1% NP40; 0.25% sodium deoxycholate; 15 mM NaCl; 1 mM EDTA; 1 mM NaF; and, Roche protease and phosphatase inhibitor tablets (Basel, Switzerland). Fluorescence-activated cell sorting (FACS) buffer and Western blot TBST buffer were similarly prepared in the laboratory (FACS: PBS (Ca^2+^/Mg^2+^ free), 1% BSA, and 0.1% sodium azide; TBST: 20 mM Tris buffer, 0.1% Tween 20, pH 7.4).

### HNSCC Cells and Cell Culture Conditions

The HNSCC radiation sensitive SCC-61, genetically matched radiation resistant rSCC-61 cells (17–21), MTHFD2 knockdown rSCC-61 cells (MTHFD2 KD rSCC-61), and the respective scramble shRNA control rSCC-61 cells (scRNA rSCC-61) were cultured in DMEM/F12 media containing 10% FBS and 1% penicillin/streptomycin at 37°C using a 5% CO_2_ incubator. The cell culture media was replaced every other day and before lysis when the cells reached 80–90% confluency. Stable MTHFD2 KD rSCC-61 and scRNA cells were generated by transfection of rSCC-61 cells with MTHFD2 shRNA and the scRNA, respectively. rSCC-61 cells were seeded in 6-well tissue culture plates at a density of 3,000 cells/cm^2^ and allowed 24 h to attach to the culture plates. When the cells reached 70–75% confluency, the cells were transfected with 50 nM MTHFD2 shRNA or 50 nM scRNA using Lipofectamine 2000 as recommended by the manufacturer's protocol and incubated for 48 hrs. The cells were then incubated with complete cell culture media (DMEM/F12, 10% FBS) containing puromycin (1 μg/mL) to facilitate the selection of MTHFD2 KD cells. The cells were further maintained in selection medium for additional 48 h resulting in stably transfected MTHFD2 KD rSCC-61 cells and the respective scRNA rSCC-61cells.

### Treatment With Ionizing Radiation and Formulation of β-Lapachone

HNSCC cells and tumors have received indicated doses of ionization radiation (IR) using a 444 TBq 12,000 Ci self-shielded ^137^Cs (Cesium) irradiator (Mark 1, Model 68A, JL Shepherd and Associates, San Fernando, CA, USA). β-Lapachone stock solution (50 mM) was prepared in DMSO and kept in 10 μL aliquots at −80°C. For the *in vivo* studies, β-lap was complexed with cyclodextrin (HPβCD) to increase solubility and bioavailability, as described previously (22).

### Cell Proliferation and Clonogenic Cell Survival Assays

Both cell proliferation and clonogenic survival assays were performed. Briefly, for proliferation assays the cells were trypsinized and ~5,000 cells/well were seeded in 96-well plates. After 24 h, the cells were treated with 2, 4, 8, and 12 μM β-lap and incubated for 2 h at 37°C in a 5% CO_2_ incubator. The culture media was then replaced with fresh complete media, and the cells were incubated for additional 24 h at 37°C/5% CO_2_ incubator. Cell proliferation was quantified using the fluorescence based CyQuant assay following the manufacturer's protocol.

Clonogenic survival assays were performed to determine the synergy with β-lap and ionizing radiation following previously reported methods (23). HNSCC cells (SCC-61, rSCC-61, MTHFD2 KD rSCC-61, and scRNA rSCC-61) were seeded at a density of 300 cells/well into 6-well plates. Cells were kept overnight at 37°C in a 5% CO_2_ incubator. The next day, the cells were irradiated (2 Gy, single dose or sham) followed by addition of the indicated concentration of β-lap or vehicle control and further incubated for 2 h. Upon completion of the incubation period, the culture media was replaced with fresh complete media and the cells were returned to the 37°C/5% CO_2_ incubator for 6–7 days. The cells were then fixed with a solution of ice-cold acetic acid: methanol (3:7) and stained with 0.5% crystal violet for 2 h at room temperature. The plates were washed with running water to remove the residual stain. Colonies containing >50 cells were counted under a light microscope and the survival fraction was calculated as described (23).

### Western Blot Analysis

HNSCC cells were lysed with modified RIPA buffer supplemented with Roche protease and phosphatase inhibitor tablets, and the protein concentration was measured using the BCA assay. An equal amount of protein (20 mg) was separated by SDS-PAGE and transferred to nitrocellulose membranes for Western blot analysis. Membranes were probed with indicated primary antibodies overnight at 4°C. Next day, the membranes were washed three times with TBST buffer and further incubated with corresponding secondary antibodies for 1 h at room temperature. The membranes were washed again three times with TBST buffer (in all cases, each TBST wash cycle was 15 min), incubated with the SuperSignal chemiluminescent HRP substrate, and the images were collected using an Amersham Imager 600 (GE Healthcare Life Sciences). Antibodies against actin, α-tubulin, or GAPDH were utilized as controls for equal loading.

### Enzyme Activity Assays

NQO1 activity in cell extracts was assayed following published protocols (5). Briefly, NADH (200 μM) used as a reducing agent (electron donor) and menadione (10 μM) as the intermediate electron acceptor were mixed in 50 mM Tris-HCl buffer (pH 7.5) reaction mixture containing 77 μM cytochrome C (Sigma, USA) and 0.14% BSA. The cell lysate was then added into the reaction mixture and the absorbance was read at 550 nm. The enzymatic activity of NQO1 was calculated as nmol cytochrome c reduced/min/μg protein.

### Cell Cycle

About 2 × 10^5^ MTHFD2 KD and scRNA rSCC-61 cells were seeded into 6-well cell culture plates, allowed to attach, incubated overnight in serum-free medium, and then irradiated (2 Gy or sham) and treated with β-lap (3 μM) or vehicle control for 2 h. After completion of the incubation period, the culture media was replaced with fresh serum-free media and the cells were incubated for another 22 h. At the end of the incubation period, the cells were washed with PBS, trypsinized, and centrifuged at 200 × g for 5 min. The cells were again washed with PBS, fixed with ice-cold 70% ethanol for 30 min at 4°C, further washed with PBS (two times), and incubated with PBS containing 100 μg/mL RNase A and 50 μg/mL propidium iodide. The cells were washed three times with PBS and resuspended into 300 μL FACS buffer and subjected to flow cytometry using a BD Accuri 6 for analysis. Data analysis to quantify the cell cycle distribution was performed with FCS Express 6 Flow software (*De Novo* Software, Pasadena, CA, USA).

### Mitochondrial Respirometry Analysis

The effects of β-lap on mitochondrial function were measured using the Seahorse Mito Stress Test following the manufacturer's protocol on a Seahorse XF24 (Agilent Technologies, Santa Clara, CA, USA). MTHFD2 KD and scRNA rSCC-61 cells (~4,000 cells/well) were seeded on a 24-well Seahorse plate and allowed to attach for 24 h. Next day, the cells were exposed to β-lap (3 μM) or vehicle control for 2 h in complete growth media, washed, and transferred to the Seahorse instrument for collection of oxygen consumption data. After baseline reading, the injections were performed as follows: (1) oligomycin (1 μM) at 27 min, FCCP (1 μM) at 51 min, and Antimycin A/Rotenone (1 μM) at 75 min. After the completion of data collection, the cells were lysed and quantified with CyQuant for data normalization. The data were analyzed with Wave software (Agilent Technologies).

### Flow Cytometry Analysis of Mitochondrial ROS and Mitochondrial Protein Oxidation

Mitochondrial ROS was detected in live cells using MitoSOX and flow cytometry analysis. Briefly, MTHFD2 KD and scRNA rSCC-61 cells were irradiated (2 Gy or sham) and further treated with β-lap (3 μM) or vehicle control for 2 h After completion of the incubation period, the cells were washed with PBS and further incubated with 1 μM MitoSOX for 30 min at 37°C in a 5% CO_2_ incubator. The cells were then washed three times with PBS, resuspended in FACS buffer and subjected to flow cytometry using a BD FACS Canto II Cell Analyzer (BD Biosciences, San Jose, CA, USA). Similarly, to determine the mitochondrial protein oxidation, the cells were treated with radiation and β-lap as described for MitoSOX analysis, and then stained with DCP-NEt_2_C (for 30 min at 37°C). DCP-NEt_2_C contains a protein sulfenic acid reacting group and coumarin to facilitate localization to mitochondria (24). After completion of the incubation period, cells were washed with PBS, fixed with ice-cold methanol for 5 min, further washed with PBS, resuspended in the FACS buffer and analyzed using a BD LSRFortessa Flow Cytometer. Data analysis was performed with FCS Express 6 Flow software (*De Novo* Software, Pasadena, CA, USA).

### Tumor Xenograft Implant in Nude Mice

The effects of MTHFD2 KD on the anti-tumor efficacy of β-lap and ionizing radiation were investigated *in vivo* using 4–5 weeks old female nu/nu nude mice obtained from Charles River Laboratories, MA, USA. The animal studies were performed under a protocol approved by the Wake Forest University Institutional Animal Care and Use Committee and in accordance with the guidelines for ethical conduct in the care and use of animals in research. Mice were housed for 1 week after arrival at the animal facility before the start of the experiment. Mice were fed pellet diet and received water *ad libitum*. The xenograft tumor was generated subcutaneously by injecting 5 × 10^5^ MTHFD2 KD and scRNA rSCC-61 cells suspended in growth factor reduced matrigel in a single flank on the dorsal surface of mice. Once the tumor size reached minimum 100 mm^3^, the mice were randomly divided into subgroups (5 mice/group) and treated as follows: (1) HPβCD (intravenous administration); (2) ionizing radiation (2 Gy) targeted to the xenograft tumor; (3) β-lap/HPβCD complex (20 mg/kg body weight; intravenous administration); or (4) ionizing radiation (2 Gy) + β-lap/HPβCD complex (20 mg/kg; intravenous administration). Mice have received treatment every other day and tumor volume was measured using a digital caliper. Mice were euthanized after 10 days, the tumors were collected, washed with PBS, and weighted. A portion of the tumor was fixed in 10% formaldehyde and stained with hematoxylin and eosin.

### Statistical Analyses

All experiments were performed in minimum three biological replicates and data are presented as mean ± standard deviation (SD). ANOVA or Students' *t*-test was utilized for statistical analysis using SPSS 7.0 and Excel software.

## Results

### Radiation Resistant HNSCC rSCC-61 Cells Display Increased Sensitivity to β-Lapachone

**Figure 1:**
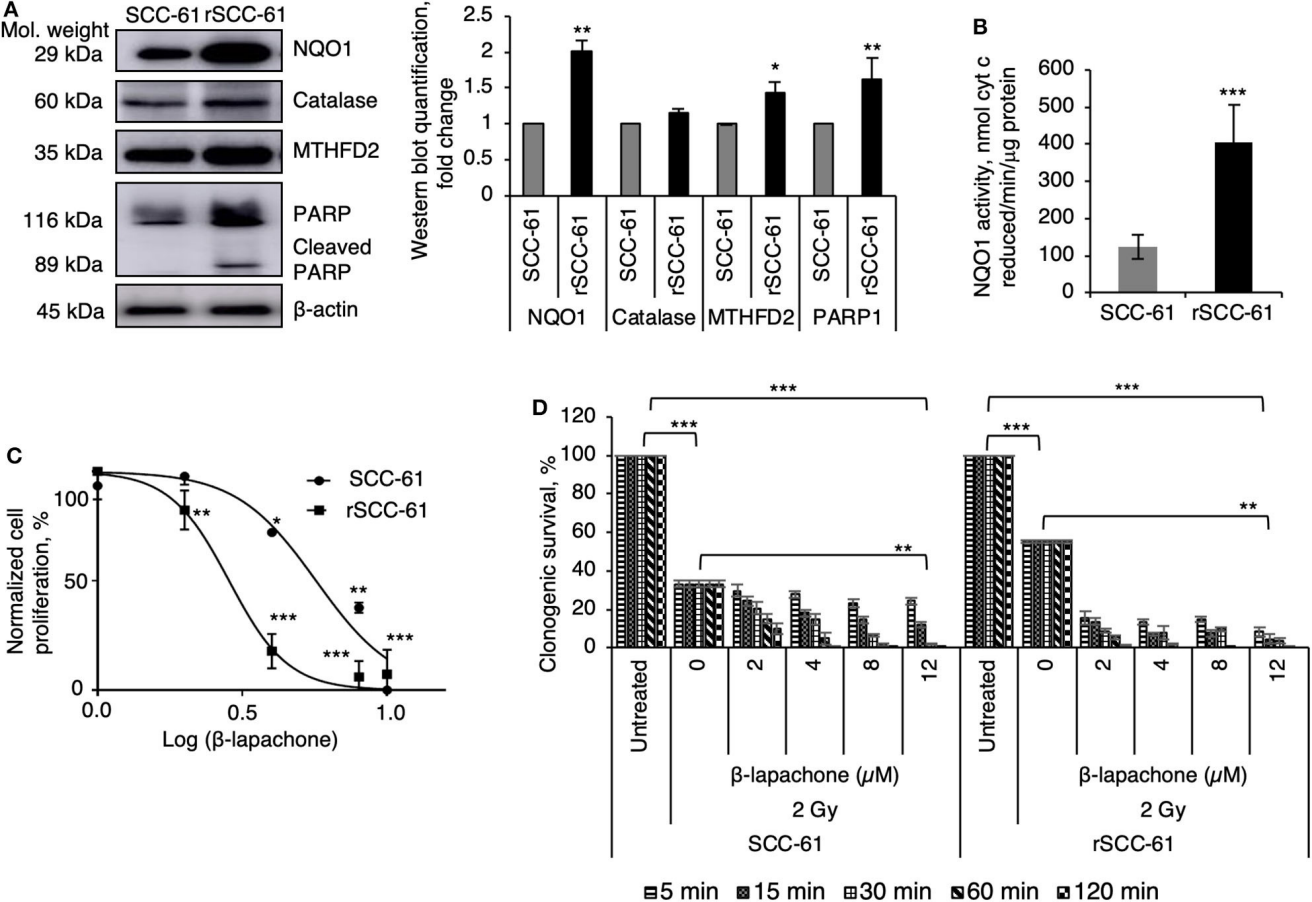
Radiation resistant rSCC-61 cells have increased sensitivity to β-lapachone. **(A)** Western blot analysis of radiation sensitive SCC-61 and radiation resistant rSCC-61 cell lysates show approximately equal expression of catalase and increased levels of NQO1, MTHFD2, and cleaved PARP1 in rSCC-61 cells. The quantification of Western blots is presented in the right panel (*n* = 3; Student's *t*-test *p 0.01–0.05, **p 0.001–0.01 relative to SCC-61). **(B)** Measurement of NQO1 activity validate the findings from Western blot analysis showing ~4-fold higher activity in rSCC-61 cells (*n* = 3; ***p < 0.001). **(C)** Cellular proliferation of SCC-61 and rSCC-61 cells treated with 0, 2, 4, 8, 12 μM β-lap demonstrate increased sensitivity of rSCC-61 cells to β-lap (*n* = 3, One-Way ANOVA with multiple comparisons *p 0.01–0.05, **p 0.001–0.01, and ***p < 0.001 relative to the respective untreated cells). **(D)** Clonogenic survival assays demonstrate the radiation sensitizing activity of β-lap, which is dependent on both the concentration of β-lap and the time of exposure to drug. *p*-values are indicated on the graph (*n* = 3, One-Way ANOVA with multiple comparisons).

The initial studies focused on the evaluation of previously reported biomarkers underlying the mechanisms of β-lap cytotoxicity (NQO1, catalase, PARP1) and MTHFD2 in the SCC-61 and rSCC-61 HNSCC cells (9). The Western blot analysis in Figure 1A show statistically significant increased expression of NQO1, MTHFD2, and PARP1, and slight but not statistically significant difference in the catalase levels (*p* > 0.05) in rSCC-61 relative to SCC-61 cells, suggesting potentially higher sensitivity to β-lap in rSCC-61 cells driven by increased NQO1 to catalase ratio. The activity of NQO1 was also significantly higher in rSCC-61 cells corroborating the Western blot analysis (*p* < 0.001, Figure 1B). To further investigate if the differences in the NQO1 and catalase profiles translated in increased sensitivity of rSCC-61 cells to β-lap, we performed *in vitro* cell proliferation assays. The data confirmed the anticipated increased sensitivity of rSCC-61 to β-lap (IC50 2.8 μM) compared with SCC-61 [IC50 5.7 μM) (Figure 1C). As radiation therapy is being used as first line treatment for HNSCC, and β-lap was shown previously to sensitize cells to radiation treatment (9)], we sought to quantify the impact of combined radiation and β-lap on clonogenic survival of SCC-61 and rSCC-61 cells. The cells were irradiated with 2 Gy and immediately treated with increasing doses of β-lap for 5 min to 2 h time intervals as noted in Figure 1D. As expected, in the absence of β-lap the extent of radiation-induced inhibition of clonogenic survival was greater in the radiation sensitive SCC-61 cells than in the radiation resistant rSCC-61 cells, constituting an important internal control for the assay. Addition of β-lap further decreased survival of SCC-61 in both time and dose-dependent manner (*p* < 0.01), but the cytotoxic efficacy in rSCC-61 cells was notably stronger across conditions (*p* < 0001).

### MTHFD2 Knockdown Sensitizes rSCC-61 Cells to Radiation and β-Lapachone Treatment

**Figure 2:**
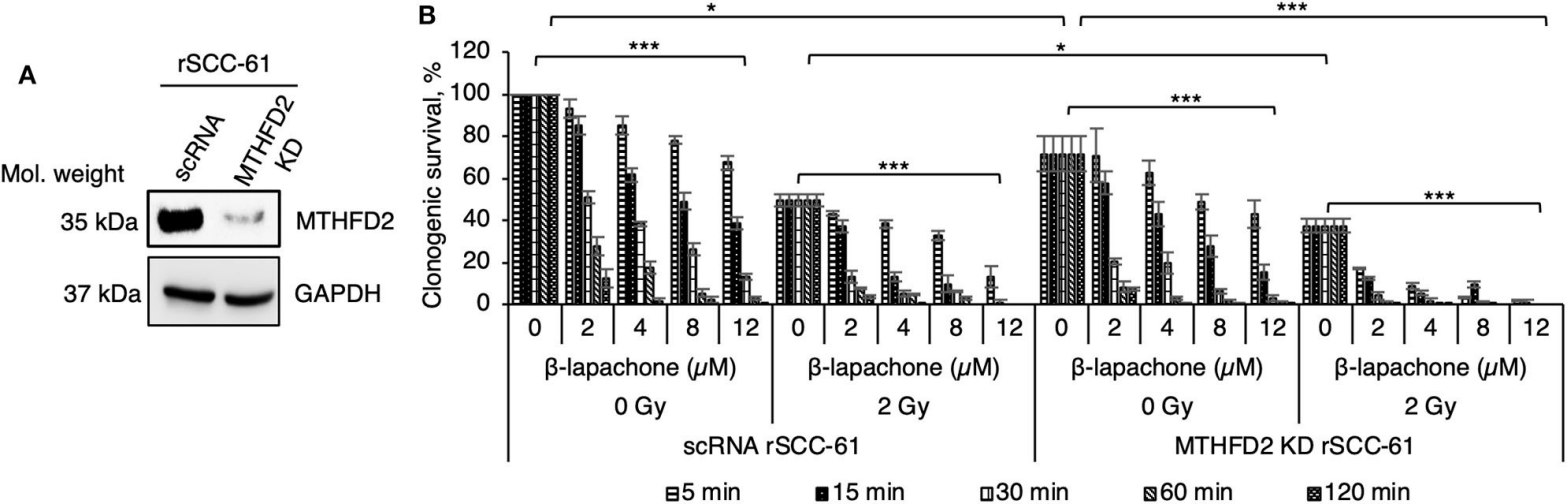
MTHFD2 depletion in rSCC-61 increases β-lapachone cytotoxicity and sensitizes cells to radiation therapy. **(A)** Western blot analysis of scRNA control and MTHFD2 KD rSCC-61 confirms depletion of MTHFD2 in rSCC-61 cells transfected with MTHFD2 shRNA. **(B)** Clonogenic survival assays demonstrate increased sensitivity to both β-lap and ionizing radiation in MTHFD2 KD rSCC-61, which is dependent on both the concentration of β-lap and the time of exposure to drug. The maximum suppression of clonogenic survival was achieved in MTHFD2 KD rSCC-61 cells treated with combined ionizing radiation and β-lap. *n* = 3, One-Way ANOVA with multiple comparisons *p 0.01–0.05 and ***p < 0.001.

Our previous computational analysis identified mitochondrial MTHFD2 as a major contributor to NAD(P)H production (20, 21). Indeed, the data in Figure 1A show higher level of MTHFD2 expression in the radiation resistant rSCC-61 cells compared to SCC-61 cells. To further investigate the role of MTHFD2 in the response of rSCC-61 cells to β-lap and ionizing radiation, we generated MTHFD2 KD and scRNA control rSCC-61 cells using shRNA technology (Figure 2A). Irradiated or sham-irradiated MTHFD2 KD and scRNA rSCC-61 cells were treated with increasing concentrations of β-lap (2–12 μM) or vehicle control for different time intervals (5 min−2 h) and the survival fraction was quantified for each treatment condition. As shown in Figure 2B, the clonogenic survival of MTHFD2 KD rSCC-61 cells was lower than scRNA rSCC-61 across treatment conditions. MTHFD2 KD rSCC-61 cells showed greater sensitivity to radiation treatment compared to scRNA rSCC-61 (*p* < 0.05), and treatment with β-lap resulted in more pronounced dose and time-dependent increases in cell death in MTHFD2 KD rSCC-61 as compared to scRNA rSCC-61 cells.

### Combined β-Lapachone and Radiation Treatment Induces DNA Damage and Increases Mitochondrial ROS and Protein Oxidation in MTHFD2 Deficient rSCC-61 Cells

**Figure 3:**
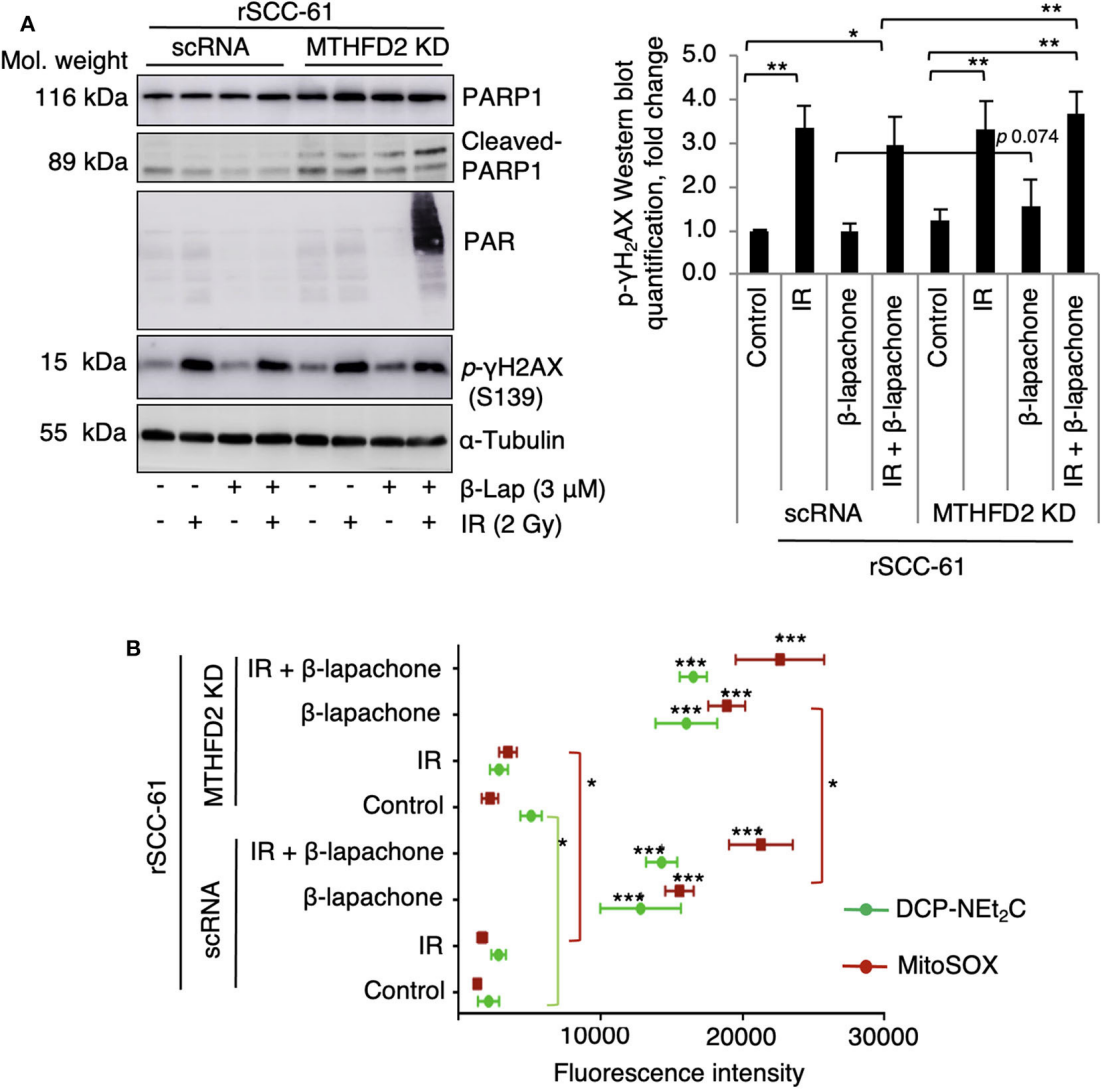
Combination of radiation and β-lapachone treatment induces mitochondrial ROS and DNA damage in MTHFD2 KD rSCC-61. **(A)** Western blot analysis of biomarkers of DNA damage in control scRNA and MTHDF2 KD rSCC-61 cells exposed to radiation (IR, 2 Gy) and β-lap (3 μM, 1 h). The blots were probed with antibodies against phosphorylated γH_2_AX (pS139), PARP1, cleaved PARP1, and protein PARylation. α-Tubulin antibodies were used as loading control. Quantification of phosphorylated γH_2_AX Western blot data is shown on the right panel. *n* = 3, Student's *t*-test *p 0.01−0.05, and **p 0.001–0.01. **(B)** Flow cytometry analysis of mitochondrial oxidative state in control scRNA and MTHFD2 KD rSCC-61 cells treated as in *Panel A* and further stained with MitoSOX and DCP-NEt_2_C probes to detect mitochondrial superoxide and mitochondrial protein sulfenylation, respectively (*n* = 3; Student's *t*-test ***p < 0.001 relative to untreated scRNA rSCC-61 cells, *p 0.01–0.05 indicate statistically significant effects of MTHFD2 KD for control and treatment conditions).

NQO1-dependent β-lap induced cell death has been reported to be the result of increased DNA damage initiated by the massive generation of ROS. To evaluate the consequence of MTHFD2 KD on the sensitivity of rSCC-61 cells to ionizing radiation and β-lap - induced DNA damage, MTHFD2 KD and scRNA rSCC-61 cells were irradiated followed by treatment with β-lap (3 μM, 1 h). Western blot analysis shown in Figure 3A indicates a significant amount of protein PARylation induced by combined radiation and β-lap treatment but only in MTHFD2 deficient rSCC-61 cells. Cleaved-PARP1 was also relatively increased upon treatment with radiation and β-lap in MTHFD2 KD rSCC-61 cells. Phosphorylation of γH_2_AX was significantly higher in irradiated cells regardless of MTHFD2 status or β-lap treatment (*p* 0.001–0.01). The MTHFD2 KD rSCC-61 cells show slightly more DNA damage when treated with β-lap alone (*p* 0.074) or in combination with radiation (*p* 0.004) as reflected indirectly by the phosphorylation status of γH_2_AX (Figure 3A).

Given the predominant mitochondrial localization of MTHFD2, we quantified next the effects of MTHFD2 depletion on mitochondrial ROS using MitoSOX and mitochondrial protein oxidation with DCP-NEt_2_C, a selective fluorescent probe targeting protein sulfenylation (24). As shown in Figure 3B, MTHFD2 knockdown or radiation induced only minor changes in mitochondrial ROS or protein sulfenylation (either not significant or *p* 0.01–0.05). However, treatment with β-lap alone or in combination with radiation induced significantly higher levels of both mitochondrial ROS and protein sulfenylation, which was augmented by MTHFD2 depletion.

### MTHFD2 Knockdown Enhances Mitochondrial Toxicity of β-Lapachone in rSCC-61

**Figure 4:**
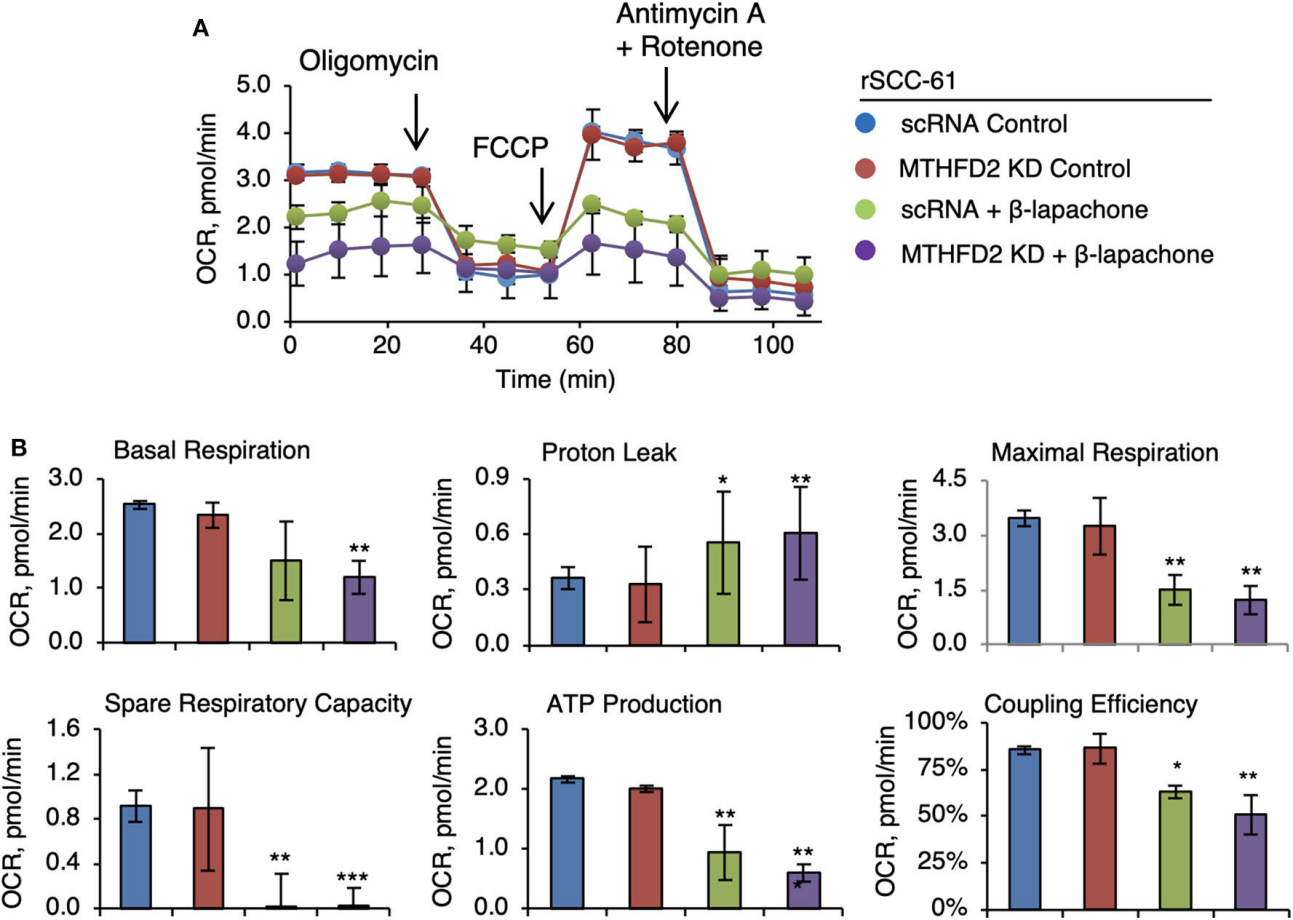
MTHFD2 depletion does not significantly impact mitochondrial respiration but cooperates with β-lapachone to decrease ATP production. **(A)** Mitochondrial respirometry analysis in control scRNA and MTHFD2 KD rSCC-61 cells pre-treated with β-lap (3 μM, 2 h) or the respective vehicle control. **(B)** Analysis of mitochondrial respirometry parameters indicate significant effects of combined MTHFD2 knockdown and β-lap treatment on the basal respiration, coupling efficiency, ATP production and H^+^-leak. Treatment with β-lap alone completely depleted the spare respiratory capacity (*n* = 3; Student's *t*-test *p 0.01–0.05, **p 0.001–0.01, and ***p <0.001 calculated relative to scRNA rSCC-61 cells).

Earlier studies have established the effects of β-lap on mitochondrial respiration (7, 25) and the results in Figure 3B corroborate the findings in these publications. Given the function of MTHFD2 in mitochondrial NAD(P)H production, we hypothesized a more drastic collapse of mitochondrial respiration in MTHFD2 deficient cells. Therefore, we set up to determine the effects of β-lap on mitochondrial respiration by measuring the oxygen consumption rate in MTHFD2 KD and scRNA rSCC-61 cells using the standard Seahorse Mito Stress test (Figure 4). As anticipated, β-lap treatment decreased basal respiration in both MTHFD2 KD and scRNA rSCC-61 cells but achieved statistical significance only in MTHFD2 depleted cells. Similarly, the effect of β-lap treatment on all other parameters of mitochondrial respiration was augmented by MTHFD2 depletion with the exception of spare respiratory capacity, which was completely obliterated with β-lap treatment regardless of MTHFD2 status.

### MTHFD2 Knockdown Does Not Significantly Impact Cell Cycle Distribution in rSCC-61 Cells

**Figure 5:**
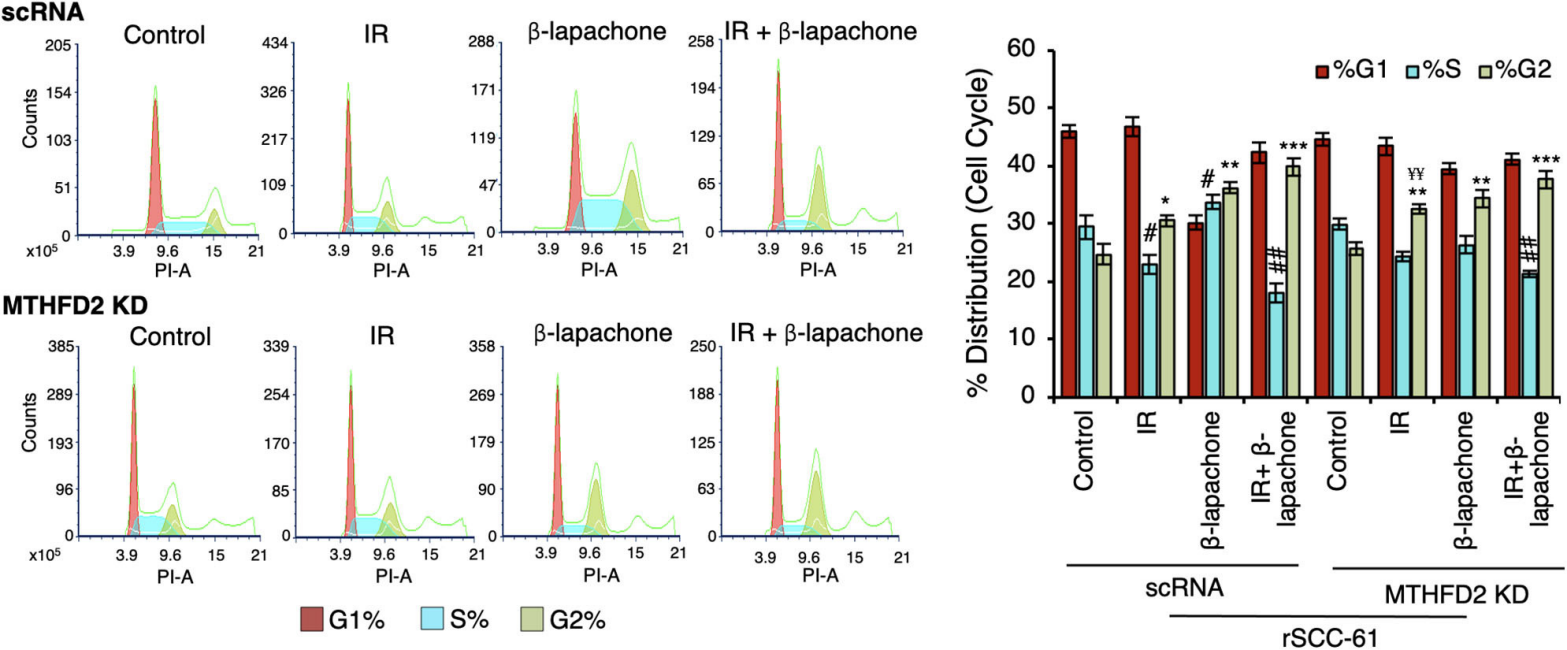
MTHFD2 knockdown significantly alters the effects of β-lapachone on cell cycle progression. Flow cytometry analysis of control scRNA and MTHDF2 KD rSCC-61 cells exposed to radiation (IR, 2 Gy) and β-lap (3 μM, 2 h) and stained with propidium iodide for cell cycle analysis shows comparable shifts in cell cycle distribution in control and MTHFD2 depleted cells under all treatment conditions with the exception of β-lap alone (*n*=3; One-Way ANOVA with multiple comparisons *p 0.01–0.05, **p 0.001–0.01, and ***p < 0.001 calculated relative to %G2-phase of vehicle-treated scRNA rSCC-61 cells; ^#^*p* 0.01–0.05, ^##^*p* 0.001–0.01 calculated relative to %S-phase of vehicle-treated scRNA rSCC-61 cells; and, ^¥¥^*p* 0.001–0.01 relative to radiation treated scRNA rSCC-61).

Both treatment with ionizing radiation and β-lap are known to induce cell cycle arrest at G1/S and G2/M checkpoints (18, 26, 27). In order to investigate the contribution of MTHFD2 to these effects, the cell cycle distribution was analyzed side-by-side in MTHFD2 KD and scRNA rSCC-61 cells treated with β-lap (3 μM, 2 h), ionizing radiation (2 Gy), and their respective vehicle and sham-irradiation controls. As shown in Figure 5, MTHFD2 depletion did not alter cell cycle distribution in rSCC-61 cells and as expected, ionizing radiation increased the proportion of cells in G2 phase in both scRNA control and MTHFD2 KD cells. This is consistent with well-established mechanisms of radiation resistance preventing cells with DNA damage from entering mitosis. Interestingly, while β-lap promoted cell cycle progression from G1 to S-phase, MTHFD2 knockdown prevented this activity, and the cell cycle distribution of MTHFD2 KD rSCC-61 cells treated with β-lap resembled the profile of radiation-treated rSCC-61 cells (scRNA or MTHFD2 KD). Overall, combined treatment with ionizing radiation and β-lap shows a statistically significant decreased distribution of cells in S-phase and increased G2-phase compared to untreated cells, regardless of MTHFD2 status.

### MTHFD2 Knockdown Suppresses rSCC-61 Tumor Growth and Increases Sensitivity to Radiation

**Figure 6:**
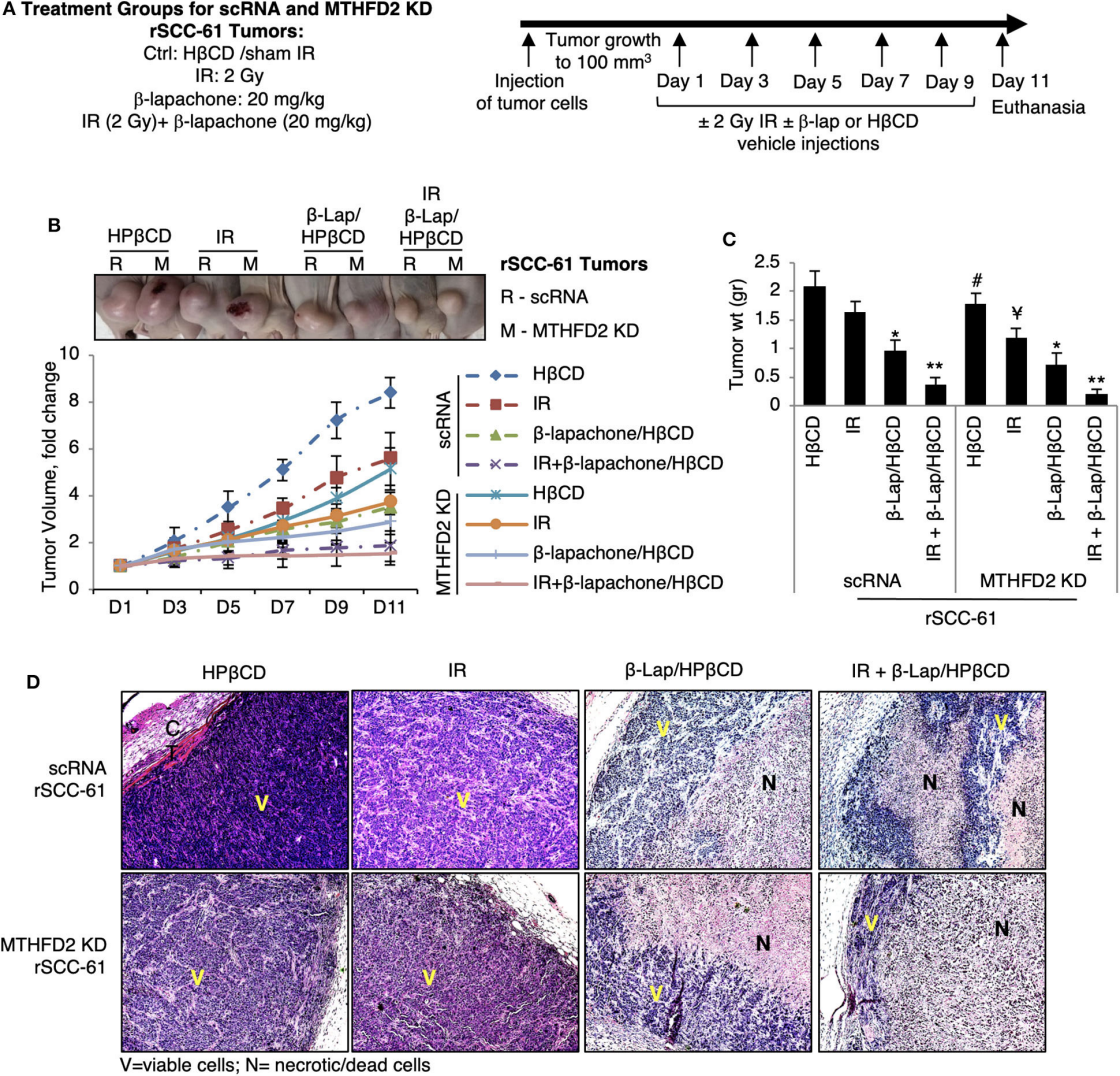
*In vivo* study of combined antitumor activity of β-lapachone with radiation in HNSCC xenograft mice. **(A)** Description of treatment groups and experimental design. **(B)** Athymic mice (nu/nu) bearing 100 mm^3^ scRNA or MTHFD2 KD rSCC-61 tumor xenografts were randomly divided into subgroups (5 mice/group) to receive treatment as described in Materials and Methods. Data represent the fold change in the mean value of tumor volume (mean ± SD). The images at the top represent the tumors at Day 11. **(C)** Comparison of tumor weight measured after euthanasia at Day 11 (*n* = 5; * represent comparisons with the respective untreated tumors in each group, scRNA and MTHFD2 KD rSCC-61, respectively; Student's *t*-test *p 0.01–0.05, **p 0.001–0.01; ^#^*p* 0.01–0.05 represents the comparison of untreated MTHFD2 KD and scRNA rSCC-61 tumors, and ^¥^*p* 0.01–0.05 represents the comparison of radiation treated MTHFD2 KD and scRNA rSCC-61 tumors). **(D)** Representative H&E images of tumors isolated from the eight animal groups. IR, ionizing radiation; V, viable cells; N, necrotic/dead cells.

To further investigate the consequence of MTHFD2 knockdown on the response to ionizing radiation and β-lap, we performed *in vivo* studies using subcutaneous scRNA and MTHFD2 KD rSCC-61 xenograft tumors in nu/nu nude mice (Figure 6A). When the tumor reached minimum 100 mm^3^, the mice were randomly divided into subgroups for treatment. The results summarized in Figure 6B, show significantly decreased tumor growth in MTHFD2 KD rSCC-61 tumors compared with scRNA rSCC-61 controls by ~50%. As expected based on the results of the cell culture studies, the scRNA rSCC-61 tumors were more sensitive to β-lap than to ionizing radiation, and the combined ionizing radiation and β-lap treatment (2 Gy followed by β-lap 20 mg/kg body weight) resulted in an almost complete suppression of tumor growth irrespective of MTHFD2 status. It is possible that the contribution of MTHFD2 noted in the cell culture studies (Figure 2) is masked in these experiments by the strong activity of combined β-lap and radiation treatment. However, depletion of MTHFD2 significantly sensitized the tumors to radiation treatment and slightly improved the efficacy of β-lap (Figure 6C). At the end of the experiment, the xenograft tumors were isolated, fixed and stained with hematoxylin and eosin (H&E, Figure 6D). While treatment with ionizing radiation did not significantly alter the morphology of either scRNA or MTHFD2 KD rSCC-61 tumors, treatment with β-lap caused suppression of tumor vascularization in both scRNA and MTHFD2 KD rSCC-61 tumors. Necrosis was noted in the core of both tumor types; however, necrosis was higher in the MTHFD2 knockdown tumors. In the groups receiving combined radiation and β-lap treatment, there was more extensive necrosis occurring in the central and peripheral areas of scRNA rSCC-61 tumors, and this was increased in MTHFD2 KD rSCC-61 tumors showing large centralized and peripheral necrosis in the entire tumor.

## Discussion

Clinical management of HNSCC presents with multiple treatment challenges limiting overall survival rates and patients' quality of life. Amongst these, resistance to radiation or chemoradiation treatment constitutes a significant problem and new approaches are needed to improve the response to standard of care therapies and to prevent the associated damage to normal tissues. Currently, Federal Drug Administration approved the use of four chemotherapeutics (methotrexate, bleomycin, docetaxel, and hydroxyurea), one targeted therapy (Cetuximab, monoclonal antibody against EGFR), and one drug combination (docetaxel, cisplatin, and 5-fluorouracil) for treatment of HNSCC. Cetuximab received FDA approval in 2007 and is used as radiation sensitizer in patients with locally advanced HNSCC. However, despite the broad overexpression of EGFR in HNSCC, only 10–15% of patients respond to Cetuximab, emphasizing the need for new approaches to treat radiation resistant HNSCC (28).

β-Lapachone and its derivatives have emerged as a lead class of quinone-based NQO1 bioactivatable therapeutics and radiation sensitizers for numerous cancers (6, 7). Traditionally, the activity of β-lap has been linked to NQO1 expression and the NQO1 to catalase ratio, a higher ratio indicating increased capacity of ROS accumulation and cell death as ROS production outpaces degradation to non-toxic products (e.g., H_2_O_2_ dismutation by catalase to H_2_O and O_2_). Indeed, treatment with ionizing radiation was shown to induce expression of NQO1 and higher levels of NQO1 are found in radiation resistant tumors (16), emphasizing the concept of combined radiation and β-lap treatment as an approach for treatment of radiation resistant tumors. The data presented in Figure 1 support this core principle and show increased sensitivity to β-lap in radiation resistant rSCC-61 cells expressing higher levels of NQO1 but comparable catalase relative to the matched radiation sensitive SCC-61 cells. These findings are also consistent with previously published data showing radiation sensitizing activity of β-lap in NQO1 overexpressing SqCC/Y1 HNSCC tumors (9, 21). However, despite these promising results, the translation to clinic has been challenging [e.g., ARQ761 phase I/II clinical trials (Gerber et al., 2018)] limited in part by the lack of knowledge of the molecular factors driving the efficacy of β-lap or ARQ761. As the chemotherapeutic activity of β-lap is intrinsically linked to the availability of NAD(P)H, and our prior dynamic flux balance analysis identified mitochondrial MTHFD2 as a key driver of NAD(P)H in HNSCC, including the SCC-61/rSCC-61 cells (20, 21), we sought to investigate here the contribution of MTHFD2 to the efficacy of response to β-lap when used alone or combined with ionizing radiation. The focus on MTHFD2 was also driven by the clinical precedent of folate inhibitors for treatment of HNSCC, and key publications showing increased MTHFD2 expression in rapidly proliferating solid tumors compared to normal tissue (29), MTHFD2-mediated folate metabolism playing a pivotal role in the progression and metastasis of several cancer types (30–32) with evidence that this might occur independent of its enzymatic activity (33), MTHFD2 function in purine and pyrimidine biosynthesis, critical metabolites for DNA synthesis and DNA damage repair (34), and evidence of additional nuclear localization of MTHFD2 at DNA synthesis sites (33). In the phenotypically matched HNSCC cells utilized here, the radiation resistant rSCC-61 cells showed significantly increased expression of both NQO1 and MTHFD2, thus enabling investigations of the crosstalk between these enzymes in determining the radiation sensitizing activity of β-lap.

Indeed, the *in vitro* and *in vivo* data (Figures 2, 6) show statistically significant suppression of clonogenic survival and tumor growth for rSCC-61 cells depleted of MTHFD2, and radiation sensitizing effects of either MTHFD2 knockdown or β-lap treatment. MTHFD2 depletion significantly increased mitochondrial protein oxidation, augmented the mitochondrial ROS induced by either radiation or β-lap treatment, and increased the DNA damage (pS139 γH_2_AX) when cells were treated with β-lap alone or in combination with radiation (Figure 3). Overall, treatment with β-lap drastically increased mitochondrial ROS and protein oxidation in both scRNA and MTHFD2 KD rSCC-61 cells, but induced a much lesser activation of DNA damage response (pS139 γH_2_AX), completely opposite compared to radiation, which strongly induced DNA damage and had a lesser effect on mitochondrial ROS and protein oxidation. This activity pattern in itself may explain the enhanced anti-tumor effects of combined radiation and β-lap treatment.

Interestingly, MTHFD2 KD alone did not impact mitochondrial respiration likely due to its dominant effects being exerted on NADPH and/or due to replenishing of mitochondrial NADH by the malate-aspartate shuttle. On the other hand, β-lap significantly decreased mitochondrial basal respiration, ATP production, and completely obliterated the spare respiratory capacity in these cells (Figure 4), effects further increased by MTHFD2 depletion. MTHFD2 knockdown also increased cleaved PARP1, though interestingly significant protein PARylation is observed only with combined radiation and β-lap treatment in MTHFD2 KD rSCC-61 and not in the scRNA control cells (Figure 3). MTHFD2 depletion did not significantly impact the cell cycle progression in rSCC-61 cells, consistent with findings in HeLa cells (33) but contrary to reports showing cell cycle G1/S arrest in colorectal cancer cells (35). Combination of radiation with β-lap treatment enhanced G2/M arrest in rSCC-61 cells irrespective of MTHFD2 status, but MTHFD2 knockdown prevented the accumulation of cells in S-phase induced by β-lap treatment (Figure 5). Thus, MTHFD2 knockdown in rSCC-61 cells treated with β-lap display the G1/S arrest phenotype noted in colorectal cancer cells. Clearly, more studies are needed to follow-up on these intriguing findings and elucidate the function of MTHFD2 in regulation of cell cycle and the relationship with the response to β-lap and radiation treatment. The tumor-selective cytotoxic mechanism of β-lap was further confirmed *in vivo* using xenograft animal models of radiation resistant HNSCC, which revealed cooperative mechanisms of tumor suppression and extensive necrosis in MTHFD2 knockdown tumors treated with combined β-lap and ionizing radiation (Figure 6).

In conclusion, the data presented here point to potential mechanisms of interorganelle communication connecting mitochondrial sources of NAD(P)H, cytosolic NQO1 activity, nuclear DNA damage, and cell cycle regulation. The findings are important given the increased interest in targeting mitochondrial metabolism for cancer therapies, and the ongoing efforts for both the development of MTHFD2 inhibitors and of more potent derivatives of β-lap. Considering the mitochondrial subcellular location of MTHFD2 and the cytosolic location of NQO1, an important direction for future studies is to investigate the contribution of physical and functional partitioning of NADH and NADPH between and within subcellular compartments, in particular given the dual pro- and antioxidant function of NADPH (e.g., supporting ROS production by NADPH oxidases and NQO1/β-lap, and ROS suppressing activities of thioredoxin and glutathione reductases). Importantly, our computational analysis also identified other metabolic enzymes besides MTHFD2 as significant contributors to the NAD(P)H across HNSCC tumors. Thus, the understanding of tumor heterogeneity with respect to drivers of NAD(P)H in individual cells is critical to amplify the β-lap bystander cytotoxicity and advance the clinical success of β-lap therapeutics. These studies will require use of mixed tumor cells and patient-derived xenografts, which fortunately are becoming increasingly available. This research would also benefit from development of chemical tools and methods for selective imaging of ROS and/or their oxidation products to more robustly quantify the build-up and resolution of these species upon treatment with redox-altering chemotherapeutics like β-lap *in vivo*.

## Data Availability Statement

The datasets generated for this study are available on request to the corresponding author.

## Ethics Statement

The animal study was reviewed and approved by Institutional Animal Care and Use Committee (IACUC) at Wake Forest School of Medicine.

## Author Contributions

KS performed the majority of studies presented here and was assisted by NS, who performed parallel assays of NQO1 activity to confirm the results. JEL and MLK guided the selection of MTHFD2 as target of NAD(P)H metabolism. DAB and CMF guided the experimental approach. KS prepared the initial draft of the manuscript, which was edited and finalized by AWT, MLK, and CMF. DAB reviewed the data before his passing in November 2019. All authors contributed to the article and approved the submitted version.

## Conflict of Interest

CMF holds patents on detection of protein sulfenylation and is co-founder of Xoder Technologies, LLC, which provides consulting services and commercializes reagents for redox investigations. The remaining authors declare that the research was conducted in the absence of any commercial or financial relationships that could be construed as a potential conflict of interest.
